# Applied olfactory cognition

**DOI:** 10.3389/fpsyg.2014.00873

**Published:** 2014-08-12

**Authors:** Gesualdo M. Zucco, Benoist Schaal, Mats J. Olsson, Ilona Croy

**Affiliations:** ^1^Department of General Psychology, Faculty of Medicine, University of PadovaPadova, Italy; ^2^Centre Européen des Sciences du Goût, CNRSDijon, France; ^3^Division for Psychology, Department of Clinical Neuroscience, Karolinska InstitutetStockholm, Sweden; ^4^Department of Clinical Neurophysiology, University of GothenburgGothenburg, Sweden

**Keywords:** applied olfaction, cognition, everyday life, expertise, health and disease

In recent years a significant body of research has accumulated on olfaction along several lines of investigation, ranging from molecular mechanisms to the neural and cognitive processing of olfactory information, as well as to multiple influences of odors on our everyday lives. The purpose of the present Frontiers' Research Topic is to present experimental data (run in the laboratory as well as in everyday settings), reviews and methods papers on various applied or applicable aspects of olfactory cognition along with the beneficial possibilities that olfactory cognitions make possible in ameliorating different aspects of human condition.

The present Research Topic is composed of 23 articles reunited in six fields of applied olfactory cognition. The first section concerns basic studies on odor memory and attention. In the first article, Smeets and Dijksterhuis (2014) review the potency of odors to affect human behavior. In the second article, Toet and van Schaik (2013) focus on how such priming are dependent on the congruency between the odor prime and the behavior that is supposed to be affected. In the third article, Köster et al. (2014) reverse the typical view on memory as being triggered by cues of previously encountered objects and argue that odor memory in everyday life is about detecting novelty rather than pleasantness. This section ends with an overview by Larsson et al. (2014) (article fourth) on the potency of odor-cues to generate life-long autobiographical memories.

The second section reunites contributions on the acquisition and consequence of olfactory expertise which remains relatively unexplored in olfaction. Royet et al. (2013), report brain imaging studies with different types of odor experts, including: perfumers, flavorists, and oenologists (article fifth). Thereafter Sezille et al. (2014) (article sixth) investigate whether experts do perceive the pleasantness of odorants differently than non-experts. Pagliarini et al. (2013) (article seventh) study the attitudes of consumers toward wine from organically grown grapes.

The third section of the Research Topic addresses chemoreception in everyday life. In the eighth article, Thomas-Danguin et al. (2014) and his colleagues survey how everyday odors such as food flavors, perfumes, and wines convey complex information which perception depends on sophisticated processing abilities at different levels of the system. Andersson et al. (2013) turn in the ninth article to the problem of health-risk perception of chemical exposure and its interaction with distress and the ideas the receiver has about the exposure. In the tenth article Demattè et al. (2014) review the role of olfaction in food neophobia and suggest that olfaction might work as an alerting system preventing the ingestion of potentially detrimental substances.

The fourth section of the Research Topic focuses on the relationships between olfaction and emotional processes. In the eleventh article, He et al. (2014) investigates the facial expressions of emotion in response to odors. In the twelfth article, Joussain et al. (2014) show in a combined field and laboratory study the influence of odor exposure on emotional states. In the thirteenth article, Ischer et al. (2014) present a new approach to investigate how olfactory ambiences affect visual responses in virtual worlds. In the fourteen article, Seo et al. (2013) show how personality traits affect the way attitudes toward odors. Further, Schablitzky and Pause (2014) (article fifteenth) investigate the interesting link between olfactory perception and depression. Glass et al. (2014) (article sixteenth) and colleagues show how potently everyday odors can induce emotions as happiness and disgust in the perceiver, while Triscoli et al. (2014) (article seventeenth) find interesting gender difference in how liking and wanting of odors differ over time.

The fifth section concerns aspects of human reproductive life in relation with the emission and perception of body odors. Cameron (2014) starts by reviewing how pregnancy affects the perception of environmental odors (article eighteenth). Lundström et al. (2013) (article nineteenth) show how the body odor of two day-old newborns elicits activation in reward-related cerebral areas in women, regardless of their maternal status.

In the last section of the Research Topic olfaction is considered in relation with health and disease issues. Ignatieva et al. (2014) (article twentieth) hunt for a genetic explanation of interindividual variability in perceptual and emotional processing of odors. Hummel et al. (2013) (article twenty-first) give us a close-up on how the brain processes odor mixtures; while Doty and Kamath (2014) (article twenty-second) review how our olfactory abilities change across the life span. Finally, Maurage et al. (2014) (article twenty-third) point to the role of olfaction in the establishment of alcohol dependence.

We are grateful to all of the contributors for their commitment to this project and for providing new accounts of the state of the art in applied olfactory cognition. We would also like to extend our special thanks to Professor Richard J. Stevenson for writing the foreword of this book. We hope that this e-volume will help promote further research on the applied aspects of olfactory perception and cognition and attract new scientists to the field. We also hope that it will be a useful resource for colleagues and professionals dealing with the study of the chemical senses in relation with issues on human welfare in everyday setting.

## Conflict of interest statement

The authors declare that the research was conducted in the absence of any commercial or financial relationships that could be construed as a potential conflict of interest.
